# In-Column Dehydration Benzyl Alcohols and Their Chromatographic Behavior on Pyridinium-Based Ionic Liquids as Gas Stationary Phases

**DOI:** 10.3390/molecules29163721

**Published:** 2024-08-06

**Authors:** Anastasia Yu. Sholokhova, Svetlana A. Borovikova

**Affiliations:** A.N. Frumkin Institute of Physical Chemistry and Electrochemistry, Russian Academy of Sciences, 31 Leninsky Prospect, GSP-1, 119071 Moscow, Russia; borovikova7@mail.ru

**Keywords:** gas chromatography, stationary phases, pyridinium-based ionic liquids, in-column dehydration

## Abstract

At present, stationary phases based on ionic liquids are a promising and widely used technique in gas chromatography, yet they remain poorly studied. Unfortunately, testing of “new” stationary phases is often carried out on a limited set of test compounds (about 10 compounds) of relatively simple structures. This study represents the first investigation into the physicochemical patterns of retention of substituted (including polysubstituted) aromatic alcohols on two stationary phases of different polarities: one based on pyridinium-based ionic liquids and the other on a standard polar phase. The retention order of the studied compounds on such stationary phases compared to the standard polar phase, polyethylene glycol (SH-Stabilwax), was compared and studied. It was shown that pyridinium-based ionic liquids stationary phase has a different selectivity compared to the SH-Stabilwax. Using a quantitative structure–retention relationships (QSRR) study, the differences in selectivity of the two stationary phases were interpreted. Using CHERESHNYA software, the importance of descriptors on different stationary phases was evaluated for the same data set. Different selectivity of the stationary phases correlates with different contributions of descriptors for the analytes under study. For the first time, we show that in-column dehydration is observed for some compounds (mostly substituted benzyl alcohols). This effect is worthy of further investigation and requires attention when analyzing complex mixtures. It suggests that when testing “new” stationary phases, it is necessary to conduct tests on a large set of different classes of compounds. This is because, in the case of using ionic liquids as an stationary phase, a reaction between the analyte and the stationary phase is possible.

## 1. Introduction

Over the past decade, ionic liquids have gained popularity as “green solvents”, replacing traditional organic solvents for organic synthesis and catalysis [1,2]. The term “ionic liquid” (IL) is used to describe a wide class of low-melting semi-organic salts or their mixtures [3,4]. Due to the study of their diverse properties, such as thermal stability, non-volatility, and liquid state in a wide temperature range, their application fields are actively expanding.

Currently, ILs find their place in various physicochemical methods of analysis: mass spectrometry, spectroscopy, gas and liquid chromatography, etc. [5,6,7]. In gas chromatography (GC), they have come to be used as liquid polar stationary phases (SPs) for the separation of substances, primarily due to their variable selectivity and high thermal stability [6,7,8,9]. In this area, ILs offer unprecedented opportunities to extend the selectivity and temperature range of gas chromatography columns [7,10].

Various ILs are used as gas chromatographic SPs: single cation (containing a single charged organic cation), multi-cation (containing a cation with two or more charged groups), and polymeric IL [11]. Various cations are used in IL-based SPs; for instance, derivatives of quinolinium [12,13], imidazolium, pyridinium, and guanidinium [11,14] can be employed. The diversity of IL structures results in a variety of chromatographic properties and allows the selection of SPs with the desired selectivity for the target mixture. These columns are used for various separations [11,15,16,17,18].

Despite the significant advances reached in recent years in IL-based SP development, further improvement of thermal stability and selectivity is required.

A number of studies have demonstrated the capacity of selected IL-based SPs to function within a temperature range of 300–350 °C, comparable with that of standard non-polar phases [19,20].

It should be noted that columns based on ILs have a significantly lower mass spectrometric background (five times lower), leading to their greater sensitivity compared to standard SPs [21]. Such phases provide a clear advantage in the separation of substances absorbed by various specific interactions with the polar SP of the column. Studying the chromatographic behavior of various substances on IL-based SPs and establishing the patterns is an important task. Acting as a low-polar SP for non-polar compounds, they are particularly strong in retaining molecules with strong proton-donor groups [22,23,24]. These unusual properties of ILs require further investigation, as it is possible to select phases for specific analytical problems and significantly expand their applications in chromatographic separations.

In our team’s work [25], a set of data on the retention of 178 compounds of various classes of chemical substances, including aromatic and aliphatic alcohols, etc., was obtained for three IL-based SPs (containing polysubstituted pyridinium cations) and two standard phases: polar PEG (polyethylene glycol-based) and non-polar (poly(5%-diphenyl-95%-dimethyl)siloxane). The authors employed these data for a QSRR study. However, a comprehensive comparison of the selectivity of the SP was not conducted, and the physicochemical patterns of compound retention on these SPs were not considered.

The aim of this work was to identify the characteristics of the chromatographic behavior of a diverse set of compounds with varying chemical natures on two distinct SPs: an IL-based SP (containing polysubstituted pyridinium cation) and a PEG-based SP. The rationale behind the disparate selectivity of the two phases and the associated differences in chromatographic behavior observed across a wide range of compounds was elucidated with the help of QSRR.

This work is devoted to studying the retention behavior of an IL-based SP containing polysubstituted pyridinium cation. An IL-based SP was previously described in reference [26]. Unlike previous works, this work is not limited to simple test mixtures or a single group of similar compounds. Also, in this work, for the first time, a wide range of descriptors was used to describe the selectivity of the IL-based SP and to establish the patterns of retention of the studied compounds. Before our work, the properties of IL-based SPs were described using the Abraham model or other similar solvation parameter models. These approaches are based on the assumption that the free energy of interaction between the substance and the SP depends linearly on each of the individual types of interactions.

In this study, the authors discovered an unusual behavior of aromatic alcohols, namely, in-column dehydration in the one-dimensional gas chromatography mode using an IL as an SP. This phenomenon had not been previously studied but was mentioned once when using two-dimensional GC [27].

## 2. Results and Discussion

### 2.1. Chromatographic Behavior of Ionic Liquid-Based Stationary Phases

The chromatographic behavior of 178 compounds was studied on two SPs with different selectivity. This data set was previously obtained by our team and used for the QSRR study [25]. A detailed analysis of these data in this work revealed interesting retention patterns for substituted aromatic compounds. These compounds were selected for further investigation and comparison of chromatographic behavior on different SPs.

**Figure 1:**
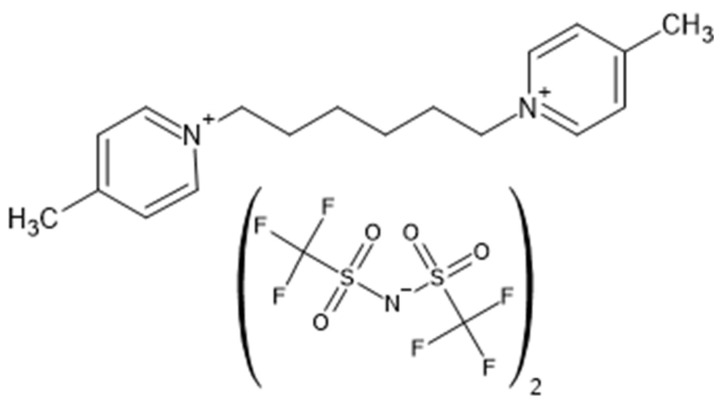
Structure of the Bis4MPyC6 ionic liquid [26].

In this work, only chromatographic retention patterns of some classes of compounds differing in the order of elution on the phase with Bis4MPyC6 (Figure 1) compared to the standard polar phase are presented.

**Figure 2:**
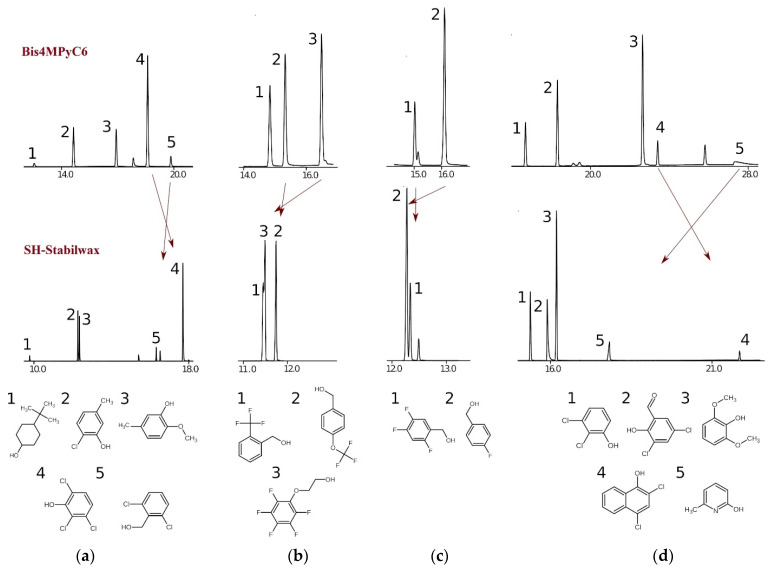
Chromatographic behavior of various analytes on the Bis4MPyC6 IL-based SP compared with the SH-Stabilwax SP. Changes in the retention order are shown by the lines (**a**–**d**). The numbers are the order of elution of the compounds.

Retention times of the considered compounds are correlated with retention times on polyethylene glycol; however, for many pairs of compounds, the elution order for an SH-Stabilwax SP and an IL-based SP is different. Some pairs of compounds co-eluting on SH-Stabilwax SP are successfully separated on IL-based SP. Several such examples are demonstrated in Figure 2. Bis4MPyC6 is more polar than SH-Stabilwax SP. It also has a suitable separation performance and provides sharp peaks.

Figure 2 shows that this SP has a different selectivity compared with SH-Stabilwax SP. Nitrogen-containing compounds demonstrate broader peaks and much stronger retention on IL-based SP compared with SH-Stabilwax SP. For instance, 6-methylpyridine-2-ol has a much higher retention than that on SH-Stabilwax SP (Figure 2d).

The difference in elution order can be explained using the QSRR study. Simple linear equations relate experimental data (chromatographic retention) and molecular descriptors (MDs) characterizing the molecules. Different contributions of the MDs lead to the expectation of different elution orders. The structure–property equations were constructed using the CHERESHNYA software (https://github.com/mtshn/chereshnya, accessed on 23 June 2024), a detailed description of which is available in [25].

The sequential addition of MDs was used. In the SEQ_ADD method, in the first step, the molecular descriptor most correlated with the retention index is selected. Using this method, ten MDs were selected for the PEG-based SP, and then a linear equation for the IL-based SP was constructed for the selected MD. The resulting equation allowed us to evaluate the importance of the descriptors. Table 1 lists the 10 most significant descriptors, from the most important to the least important (from the first descriptor to the last one, the importance decreases), describing the behavior of the PEG-based SP and IL-based SP.

Comparing the order of elution on the PEG-based SP and IL-based SP (Figure 2) under identical experimental conditions, attention is drawn to the fact that more branched and substituted aromatic alcohols are retained more strongly on the phase with the PEG-based SP than on the IL-based SP. The main contribution to the retention of such compounds on the IL-based SP is made by donor–acceptor and dispersion interactions [28]. This is also typical for columns with a Bis4MPyC6 [26], but, accordingly, to a lesser extent. And, as can be seen from the data in Table 1, for the PEG-based SP, the greater contribution is made by the FractionCSP3 descriptor (three lines in Table 1 for the PEG-based SP and ten for the IL-based SP), which is responsible for the fraction of sp^3^ hybridized carbon atoms. The descriptor PEOE_VSA11 (two lines for the PEG-based SP and eight for the IL-based SP) characterizes the accessible surface of atoms.

It is known that IL columns are characterized by high polarity, strong dipole–dipole and π-π interactions, and have a tendency to form hydrogen bonds [13,23]. As Table 1 shows, in the case of the IL-based SP, the greater contribution to the retention is made by the fr_aryl_methyl descriptor (five lines for the PEG-based SP and one for the IL-based SP), which characterizes the number of arylmethyl hydroxylation centers. As the length of the alkyl substituent in dicationic ILs increases, the donor–acceptor effect of alkyl substituents in the aromatic ring also increases.

As shown in Figure 2a, 2-chloro-5-methylphenol and 2-methoxy-5-methylphenol are practically not separated on the PEG-based SP. In this pair, the benzene ring is substituted in two positions with different substituents, but this factor does not affect the retention time. In contrast, on the IL-based SP, these compounds are well separated. Table 2 and Table 3 present the retention times, asymmetry factor, and peak resolution for the IL-based SP (Bis4MPyC6) compared with the SH-Stabilwax SP.

As can be seen in Figure 2b, 2-(trifluoromethylphenyl)methanol and 2-(pentaflurophenoxy)ethanol also does not separate on the PEG-based SP (Rs = 0.25, see Table 3), while the same pair of compounds separates well on the IL-based SP (Rs = 5.07). This fact can be explained by the strong dipole–dipole interaction of the oxygen atom in 2-methoxy-5-methylphenol and 2-(pentaflurophenoxy)ethanol with the IL-based SP. This is confirmed by the longer retention time (Rt = 22.64 min) of 2,6-dimethoxyphenol (chromatographic peak 3 in Figure 2d), which contains two methoxy groups, compared to its retention time on the standard polar phase. The same effect can also explain the strong retention of pyridine on the IL-based SP (chromatographic peak 5 in Figure 2d), where, in addition to hydrogen bonding, there is a strong dipole–dipole interaction of the lone electron pair of the nitrogen atom with the IL-based SP containing nitrogen atoms in its structure (Figure 2d). It should be noted that (4-fluorophenyl)methanol with molecular weight 126 is more strongly retained on the phase with IL than (2,4,5-trifluorophenyl)methanol with molecular weight 162 (see Figure 2c) because in the latter, there is a possible effect of screening of the hydroxo group by fluorine atoms, which weakens the interaction due to hydrogen bonding of the substance with the SP of the column.

Despite the different film thicknesses of the compared SPs, the chromatographic behavior of various compounds is not determined by this factor. When using very thin films (thinner than 0.1 μm), it is important to consider that in addition to the distribution retention mechanism, adsorption at the interphase boundary makes a significant contribution. However, with sufficient film thickness, this effect has a negligible impact on the selectivity of the SP [29]. It is also important to note that this effect significantly impacts selectivity, particularly when considering the retention of non-polar compounds on a PEG-based SP or vice versa [30]. However, in the case under consideration, this effect is less pronounced. We believe that this conclusion is accurate and that the difference in selectivity of the compared phases is not determined by the film thickness.

Thus, due to the major contribution of high polarity to various intermolecular specific interactions, which can be successfully varied in the synthesis of SP based on different ILs, it is possible to separate weakly retained or practically unretained molecules on the standard SP.

### 2.2. Dehydration of Aromatic Alcohols on the Ionic Liquid-Based Stationary Phases

**Figure 3:**
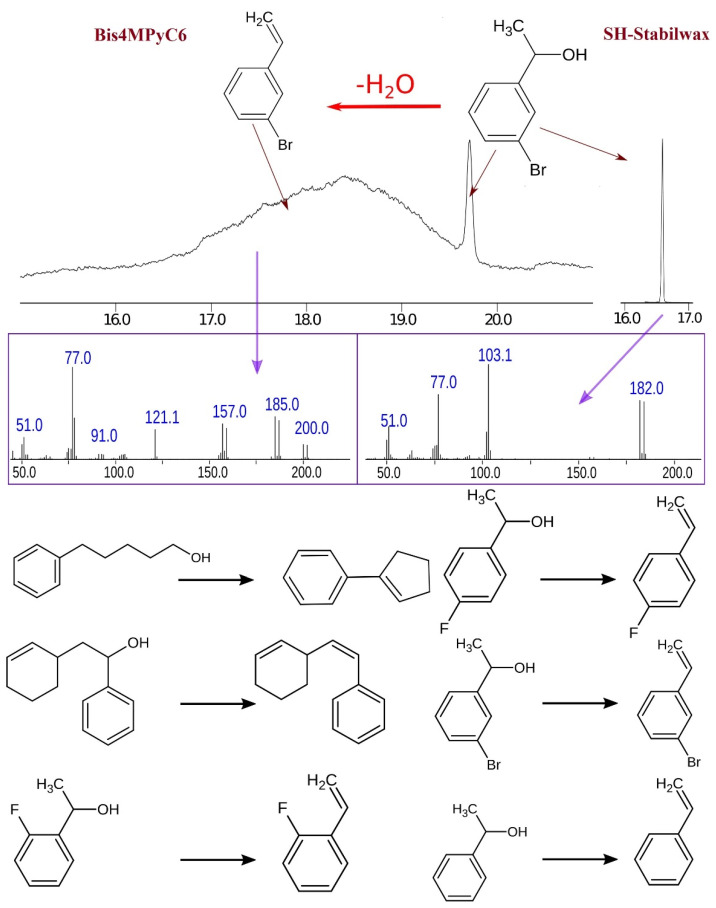
The in-column transformation of analytes on the Bis4MPyC6 IL-based SP compared with the SH-Stabilwax SP. Red arrows are the compounds corresponding to the indicated chromatographic peaks. Purple arrows indicate the mass spectrum of the corresponding compound.

For the IL-based SP, the in-column dehydration is observed for some compounds. For example, 1-(3-bromophenyl)ethanol (C_8_H_9_BrO) elutes as two peaks; the first one is very broad and corresponds to 1-bromo-3-vinylbenzene (C_8_H_7_Br) when searched in the NIST 17 database, and the second one (sharp narrow peak) corresponds to 1-(3-bromophenyl)ethanol. The same situation is observed for five other substituted derivatives of 1-phenylethanol. One more compound affected by the in-column transformation is 5-phenylpentan-1-ol. This compound undergoes simultaneous dehydration and cyclization, and 1-phenylcyclopentene is formed. Derivatives of 2-phenylethanol and any other compounds do not undergo in-column reactions. All observed in-column transformations are shown in Figure 3. The considered column continues to separate compounds well without significant changes in chromatographic behavior after many months of storage and being in operation.

A similar effect was reported by the authors [27]. The most fragile allylic alcohols identified as β-vetivol and α-isonootkatol were determined to undergo dehydration, producing, respectively, the well-known sesquiterpenes, β-vetispirene and β-vetivenene. The authors observed this phenomenon in the mode of two-dimensional GC and explained it by the fact that during modulation, when passing the hot jet of the modulator (hot jet temperature 295 °C), their dehydration occurred.

We believe that, in this case, the IL (as the SP) acts as a catalyst. A comparable outcome was observed when two alternative ILs were employed as the SP: Bis2MPyC9 and Hex4MPy (structural formulas are provided in [25]). The same compounds under similar conditions undergo a similar reaction of in-column dehydration. The catalytic properties of IL are well-known in the literature and are widely studied [31,32]. In the majority of cases, the IL in question exhibits acidic properties, including protic IL, IL with a hydrogen sulfate anion, and IL with an acidic functional group. It should be noted, however, that there are a number of studies that consider the catalytic properties of IL that do not have pronounced acidic properties. One of the reactions catalyzed by such IL is the dehydration of benzyl alcohol derivatives, which is identical to the process observed by our team [33]. Furthermore, pyridinium IL, which is non-acidic and similar in nature to the one used in this work, also exhibits catalytic properties [34]. The exact mechanism of the catalytic reaction is currently unknown and may be the subject of further research. A possible mechanism for such a catalytic reaction is mentioned in [33].

Such a phenomenon has not been previously reported in one-dimensional gas chromatography. This effect is interesting and requires attention in the analysis of complex mixtures containing benzyl alcohols and may find particularly useful applications in natural product chemistry and metabolomics. When employing IL-based SPs for the analysis of complex mixtures, it is imperative to exercise caution to ensure that the in-column dehydration product is not erroneously identified as a constituent of the analyzed mixture.

The observed effect also indicates that testing of “new” SPs should be performed on a large set of different classes of compounds. Frequently, testing of “new” SPs is conducted on a limited number of test compounds (approximately 10 compounds) with relatively simple structures. This approach is inadequate and will not permit the detection of potential reactions of the analyte when eluting through the SP.

## 3. Materials and Methods

Gas chromatography–mass spectrometry (GC-MS) analysis was carried out using Shimadzu GCMS-TQ8040. We used two chromatographic columns with IL-Bis4MPyC6 (30 m, 0.22 mm × 0.2 μm) and SH-Stabilwax (30 m, 0.25 mm × 0.1 μm, Shimadzu, Kyoto, Japan). The GC-MS system employed the following conditions: operated in electron impact mode at 70 eV; scan rate: 1666 units/s, mass range: 44–500 *m*/*z*; carrier gas flow (a constant) = 0.6 mL/min (Bis4MPyC6) and 1.0 mL/min (SH-Stabilwax), injector temperature = 250 °C, ion source temperature = 200 °C; split ratio = 1:50; injection volume = 0.5 µL; oven temperature, initial = 50 °C–240 °C at 8 °C/min (hold 15 min). The synthesis of an IL-based SP is described in reference [35].

The authors previously obtained a data set of 178 organic compounds, which was utilized in this study. A comprehensive list of the compounds is available in the online repository [36]. Furthermore, the preceding work provides comprehensive details regarding the analytical conditions employed for these compounds. A solution comprising up to three or five compounds was prepared and subjected to GC-MS analysis.

For the QSRR study, CHERESHNYA software version 0.0.2-alpha1 (https://github.com/mtshn/chereshnya, accessed on 24 June 2024) was used, a detailed description of which is available in [25]. MDs generated with the RDKit library (https://www.rdkit.org/, accessed on 24 June 2024) were used. Ten MDs were selected, the selection procedure was repeated 200 times, and 1 randomly selected molecule was excluded from the initial data set.

## 4. Conclusions

In this work, the chromatographic behavior of various classes of chemical compounds on pyridinium-based IL as a gas stationary phase was investigated. There is a significant difference in the retention behavior of the various compounds compared to the PEG-based SP (SH-Stabilwax). The considered SP is well suited for the separation of alcohols, phenols, and other oxygen-containing and halogen-containing compounds.

A comprehensive set of descriptors was employed in the QSRR study of a vast array of structurally diverse chemical substances, facilitating the interpretation of the varying selectivity of stationary phases. It was shown that more branched and substituted aromatic alcohols are retained more strongly in the phase with the PEG-based SP than in the IL-based SP. So, in the case of the IL-based SP, the greater contribution to the retention is made by the fr_aryl_methyl descriptor, which characterizes the number of arylmethyl hydroxylation centers. As the length of the alkyl substituent in dicationic IL increases, the donor-acceptor effect of alkyl substituents in the aromatic ring also increases. The contribution of descriptors was evaluated using the previously developed program CHERESHNYA (https://github.com/mtshn/chereshnya/, accessed on 24 June 2024).

In-column dehydration was observed for the IL-based SP for substituted derivatives of 1-phenylethanol. No similar effect was observed for the standard polar SP for the same compounds. It is imperative that this phenomenon be taken into account when testing “new” SP. Failure to do so may result in false identification when analyzing complex mixtures due to the effect of in-column dehydration. To ensure the accuracy of testing, a large number of chemical compounds from different classes must be included in the testing of “new” SP.

## Figures and Tables

**Table 1:** MD selected by SEQ_ADD for two SPs.

Stationary Phase	SH-Stabilwax	Bis4MPyC6
Importance of descriptor	BCUT2D_MWHI	fr_aryl_methyl
	PEOE_VSA11	BCUT2D_MWHI
	FractionCSP3	VSA_EState3
	EState_VSA2	NumRotatableBonds
	fr_aryl_methyl	Chi0n
	MaxAbsPartialCharge	Estate_VSA2
	fr_benzene	fr_benzene
	Chi0n	PEOE_VSA11
	VSA_EState3	MaxAbsPartialCharge
	NumRotatableBonds	FractionCSP3

**Table 2:** Retention times (Rt) of the studied substances and symmetry factor of chromatographic peaks for the IL-based SP (Bis4MPyC6) compared with the SH-Stabilwax SP.

Compound	Bis4MPyC6		SH-Stabilwax	
	Rt, min	As	Rt, min	As
4-tert-Butylcyclohexanol	12.55	1.11	9.78	1.16
2-Chloro-5-methylphenol	14.64	0.96	12.27	1.10
2-Methoxy-5-methylphenol	16.92	1.06	12.36	1.03
2,3,6-Trichlorophenol	18.60	0.92	17.69	1.30
(2,6-dichlorophenyl)methanol	19.84	1.07	16.32	1.00
2-(Trifluoromethylphenyl)methanol	14.84	1.04	11.45	0.83
4-(Trifluoromethoxyphenyl)methanol	15.32	1.06	11.74	0.93
2-(Pentafluorophenoxy)ethanol	16.49	1.06	11.49	1.01
(2,4,5-Trifluorophenyl)methanol	15.02	1.06	12.34	0.96
(4-Fluorophenyl)methanol	16.11	0.96	12.28	0.83
2,3-Dichlorophenol	16.69	1.00	15.38	1.20
3,5-Dichloro-2-hydroxybenzaldehyde	18.32	1.04	15.90	1.67
2,6-Dimethoxyphenol	22.64	1.13	16.20	0.98
2,4-Dichloro-1-naphthol	23.41	0.98	21.79	1.01
6-Methylpyridine-2-ol	27.34	4.00	17.69	0.84

**Table 3:** Peak resolution (Rs) of some compounds on the IL-based SP (Bis4MPyC6) compared with the SH-Stabilwax SP.

Compounds		Rs	
		Bis4MPyC6	PEG
2-Chloro-5-methylphenol	2-Methoxy-5-methylphenol	3.84	0.35
2-(Trifluoromethylphenyl)methanol	2-(Pentafluorophenoxy)ethanol	5.07	0.25
(2,4,5-Trifluorophenyl)methanol	(4-Fluorophenyl)methanol	5.28	0.50
3,5-Dichloro-2-hydroxybenzaldehyde	2,6-Dimethoxyphenol	1.70	1.13

## Data Availability

The authors used previously developed the open-source software is available online: https://github.com/mtshn/chereshnya/ (accessed on 24 June 2024).
